# TGF-β1 induces epigenetic silence of TIP30 to promote tumor metastasis in esophageal carcinoma

**DOI:** 10.18632/oncotarget.2940

**Published:** 2014-12-03

**Authors:** Fangfang Bu, Xing Liu, Jingjing Li, Shukun Chen, Xin Tong, Chunsheng Ma, Hui Mao, Fei Pan, Xiaoyan Li, Bo Chen, Liyan Xu, Enmin Li, Geng Kou, Jun Han, Shangjing Guo, Jian Zhao, Yajun Guo

**Affiliations:** ^1^ PLA General Hospital Cancer Center Key Lab, Medical School of Chinese PLA, Beijing, P.R. China; ^2^ International Joint Cancer Institute, The Second Military Medical University, Shanghai, P.R.China; ^3^ Beijing Key Laboratory of Cell Engineering & Antibody, Beijing, P.R. China; ^4^ The 150 Hospital of Chinese PLA, Luoyang, P.R.China; ^5^ Department of Biochemistry and Molecular Biology & Institute of Oncologic Pathology, Shantou University Medical College, Shantou, P.R.China; ^6^ Department of Pharmacy, Liaocheng University, Liaocheng, P.R. China

**Keywords:** TIP30, TGF-β1, methylation, epithelial-mesenchymal transition, ESCC

## Abstract

TGF-β1, a potent EMT (epithelial-mesenchymal transition) inducer present in the tumor microenvironment, is involved in the metastasis and progression of various carcinomas, including esophageal squamous cell carcinoma (ESCC). TIP30 (30kDa HIV-1 Tat interacting protein) is a putative tumor metastasis suppressor. Here, we found TIP30 was decreased in cells undergoing EMT induced by TGF-β1, an occurrence that was related to promoter hypermethylation. TGF-β1 induced *TIP30* hypermethylation via increasing DNMT1 and DNMT3A expression, which could be restored by TGF-β antibodies. In our *in vitro* and *in vivo* studies, we showed that silence of TIP30 led to EMT, enhanced migrative and invasive abilities of ESCC cells, promoted tumor metastasis in xenografted mice; alternatively, overexpression of TIP30inhibited TGF-β1-induced EMT, and metastatic abilities of ESCC cells. Mechanically, TIP30 silencing induced the nuclear translocation and transcriptional activation of β-catenin in an AKT-dependent manner, which further resulted in the initiation of EMT. Consistently, *TIP30* was frequently methylated and downregulated in ESCC patients. Loss of TIP30 correlated with nuclear β-catenin and aberrant E-cadherin expression. TIP30 was a powerful marker in predicting the prognosis of ESCC. Taken together, our results suggest a novel and critical role of TIP30 involved in TGF-β1-induced activation of AKT/β-catenin signaling and ESCC metastasis.

## INTRODUCTION

Esophageal cancer, the eighth most common cancer in the world, is composed of two main histologic types: squamous cell carcinoma (ESCC) and adenocarcinoma (EAC) [1]. The main risk factors for ESCC, the predominant type worldwide, include smoking and alcohol abuse, both inflammatory insults to the esophagus [2]. ESCC patients usually have poor prognostic outcomes that are mainly due to metastasis and recurrence after surgery. However, the molecular mechanisms underlying tumor metastasis in ESCC is poorly understood.

Metastasis is a complex process involving acquires of motility and invasiveness properties and disseminates from primary tumors. Evidence suggests that a subset of epithelial tumor cells acquire these properties by undergoing EMT, characterized by the loss of cell polarity, and gain of mesenchymal differentiation properties [3, 4]. TGF-β1, a potent EMT inducer present in the tumor microenvironment, is involved in the metastasis and progression of various carcinomas, including gastric [5], colorectal [6] and esophageal [7, 8] carcinoma. TGF-β1 induces EMT through multiple distinct signaling mechanisms [9]. In addition to its ability to activate receptor-regulated-Smad proteins, TGF-β1 can also engage non-Smad-dependent pathways, including the Wnt / β-catenin, MAPK and Notch pathways, some of which can trigger EMT programs [10-12]. However, the underlying mechanism of how TGF-β1 activates those pathways is still unclear.

TIP30, also called CC3 or HTATIP2, is a putative tumor suppressor initially identified by a differential display analysis of mRNA in highly metastatic human variant small cell lung carcinoma (v-SCLC), versus less metastatic classic small cell lung carcinoma (c-SCLC) cell lines [13]. TIP30 is down-regulated in various human tumors due to DNA methylation or posttranscriptional regulation by miR10b [14, 15]. It's down-regulation associates with metastasis or poor prognosis of human breast cancer [16], lung cancer [17], pancreas cancer [18]and hepatocellular cancer [19]. TIP30 is able to interact with Ets-1 and inhibit Ets-1-mediated transactivation of osteopontin, an important molecule for tumor metastasis in HCC [20, 21]. Recently, inhibition of the EGFR/AKT signaling pathway by TIP30 was elucidated in breast cancer and hepatocellular carcinoma. We previously reported that TIP30 is downregulated during TGF-β1-induced EMT and decreased TIP30 induces properties of EMT and tumor-initiating cells to facilitate tumor metastasis in HCC [19]. However, the precise mechanism of how TGF-β regulates TIP30 expression remains unclear. Here, we are able to characterize the methylated regulation of *TIP30* by TGF-β1, as well as the critical role of TIP30 involved in TGF-β1-induced activation of AKT/β-catenin signaling and ESCC metastasis.

## RESULTS

### TIP30 was negatively correlated with TGF-β1 in ESCC cells

**Figure 1:**
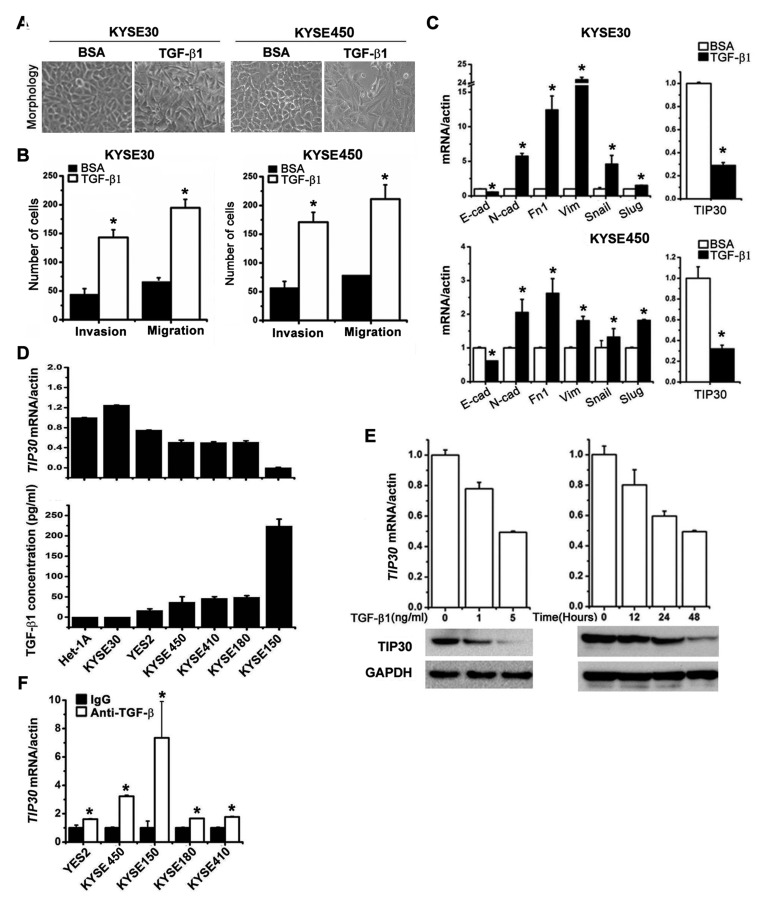
The reverse correlation of TIP30 and TGF-β1 levels in ESCC cell lines KYSE30 and KYSE450 cells were treated with 5ng/ml TGF-β1 or BSA for 48 hours, (A) morphologies of KYSE30 and KYSE450 were shown by phase-contrast microscopy (magnification, × 200); (B) invasion and migration assay were performed, total number of invaded and migrated cells were quantified and compared to the control samples; *P < 0.05; (C) the mRNA expression levels of EMT-related genes as well as *TIP30* were determined by QRT-PCR (E-cad, E-cadherin; N-cad, N-cadherin; Fn1, Fibronectin 1; Vim, Vimentin; *P < 0.05). (D) The expressions of *TIP30* mRNA were examined in 6 ESCC cell lines and a normal esophageal mucosa cell line Het-1A by QRT-PCR (upper); TGF-β1 concentrations in the cell culture supernatant were measured by specific enzyme-linked immunosorbent assay (ELISA) and normalized to the total number of cells (lower). Data are expressed as pg/ml of TGF-β1 per 10^5^ cells. (E) KYSE30 cells were stimulated with TGF-β1 at indicated concentrations or for defined intervals, and then QRT-PCR and Western blots were performed to determine the expression level of TIP30. (F) ESCC cells were treated with anti-TGF-β antibody (5ng/ml) for 3 days, and then the expression of *TIP30* mRNA was determined by QRT-PCR. Each bar represented the mean ±sd. of samples measured in triplicate, and each experiment was repeated at least three times.

TGF-β1 is a classic EMT inducer in many types of epithelial tumors, including ESCC. As shown in Fig. 1A, KYSE30 and KYSE450 cells had an epithelial-like morphology. After treatment with TGF-β1, cells underwent a morphologic change from a cobblestone-like cell morphology to a spindle-like, fibroblastic morphology, accompanied with increased cell invasion and migration ability (Fig. 1A and 1B). To better characterize TGF-β1-induced EMT, we examined the mRNA expressions of EMT-related genes and *TIP30* (Fig. 1C). We found that besides typical molecular changes of EMT, *TIP30* expression was significantly decreased upon TGF-β1 treatment in ESCC cells. To correlate the endogenous expression levels of *TIP30* with the levels of TGF-β1, we detected the mRNA expressions of *TIP30* (Fig. 1D, upper) and the secretion levels of TGF-β1 (Fig. 1D, lower) in 6 ESCC cell lines and normal esophageal mucosa cell line Het-1A. These results reveal a strong inverse correlation between *TIP30* expression and TGF-β1 level (Spearman's r=0.93, *P*=0.03). Further, qRT-PCR and Western blots showed that TGF-β1 decreased overall TIP30 expression levels in both dose-dependent (Fig. 1E, left) and time-dependent (Fig. 1E, right) manners in KYSE30 cells. In contrast, mRNA expressions of *TIP30* were restored in all silenced cell line when treated with anti-TGF-β antibody (Fig. 1F). All the above suggested that TIP30 expression was downregulated by TGF-β1 in ESCC cells.

### *TIP30* was frequently methylated and downregulated in ESCC

**Figure 2:**
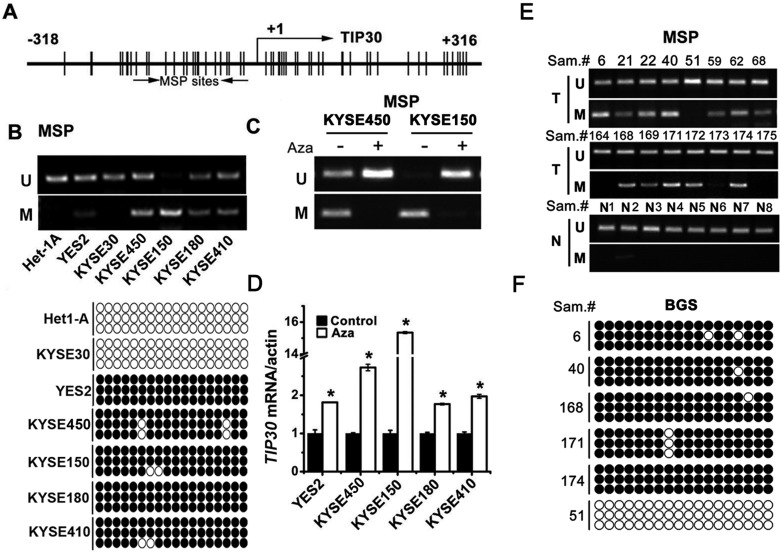
*TIP30* was frequently methylated and downregulated in ESCC (A) The CpG island region of *TIP30* is shown. The transcriptional start site is denoted as +1. The region used in MSP is underlined. (B) Methylation patterns of *TIP30* promoter were determined by MSP analysis (upper) and BGS (lower) in 6 ESCC cell lines and a normal esophageal mucosa cell line Het-1A (M, methylated; U, unmethylated; the black circle indicates the methylated cytosine while the white circle indicates the unmethylated cytosine in the dinucleotide CpG). (C) MSP analysis the methylation pattern of *TIP30* promoter in KYSE450 and KYSE150 cells after treatment with 5ng/ml of 5-Aza-2_dC for 3 days. (D) ESCC cells were treated with 5ng/ml of 5-Aza-2_dC for 3 days; the expression of *TIP30* mRNA was determined by QRT-PCR (error bar indicate SD, standard deviation; *P < 0.05). (E) Representative results of MSP analysis of *TIP30* genes in tumor tissues (T) and 8 normal esophageal mucosa tissues (N). (F) Representative results of BGS analysis of *TIP30* genes in tumor tissues. Data shown represent three different experiments.

There is a typical CpG island spanning the transcription start site of *TIP30* (Fig. 2A), as we described previously [15]. To explore whether hypermethylation of *TIP30* is involved in the decreased expression of TIP30, we examined the methylation status of *TIP30* in 6 ESCC cell lines and normal esophageal mucosa cell line Het-1A (Fig. 2B). Methylation-specific PCR (MSP) results showed that the *TIP30* promoter was unmethylated in normal esophageal mucosa cell Het-1A and KYSE30 cells which had abundant *TIP30* mRNA expression. In contrast, *TIP30* was completely methylated in KYSE150 cells, which had undetectable *TIP30* expression. Partial methylation of *TIP30* was found in the remaining ESCC cells, which had both methylated and unmethylated alleles. To confirm the MSP results, we further examined *TIP30* promoter methylation by conducting bisulfite genomic sequencing (BGS) analysis of 18 individual CpG sites within its CpG island (Fig. 2B lower). The result revealed that promoter of TIP30 was frequently methylated in ESCC cells. ESCC cell lines with methylated *TIP30* were treated with DNA demethylating agent 5-Aza-2′dC, and then MSP and QRT-PCR were performed. The results showed that treatment with 5-Aza-2′dC decreased the methylated MSP products (Fig. 2C) and increased *TIP30* mRNA expression (Fig. 2D). Together, these data demonstrate that hypermethylation of CpG islands results in epigenetic silence of *TIP30* in ESCC cell lines.

To investigate the methylation status of *TIP30* in human ESCC specimens, MSP was performed in 85 cases of ESCC tissues (T) and 8 cases of normal esophageal mucosa tissues (N, Fig. 2E). The methylation of *TIP30* was 62/85 (72.9%) in the tumor tissues and only 1/8 (12.5%) in the normal esophageal mucosa tissues. The methylation status of *TIP30* was further confirmed by BGS (Fig. 2F). The results indicate that *TIP30* is frequently hypermethylated in ESCC specimens.

### TGF-β1 promoted *TIP30* methylation through inducing DNMTs expression

**Figure 3:**
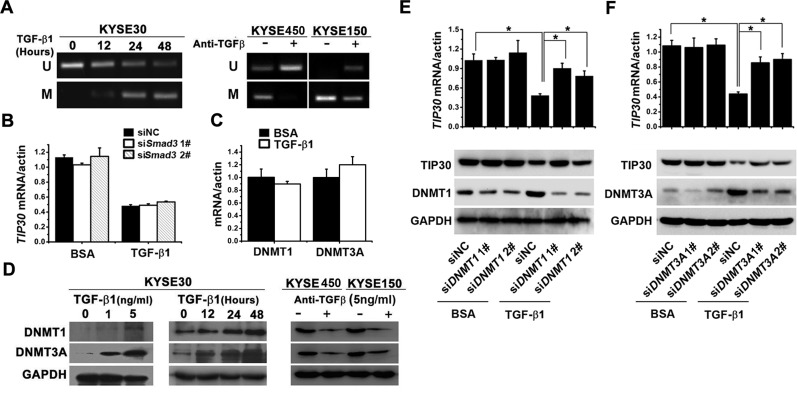
TGF-β1 promoted *TIP30* methylation through inducing DNMTs expression (A) KYSE30 cell were treated with TGF-β1 (5 ng/ml) for defined intervals, and KYSE450/KYSE150 cells were treated with anti-TGF-β antibody (5 ng/ml) for three days, then the methylation pattern of *TIP30* promoter is determined by MSP analysis. (B) KYSE30 cells transfected with siRNA against Smad3, and then treated with or without TGF-β1, 48 hours later QRT-PCR were performed to determine the expression of *TIP30*. (C) KYSE30 cells were treated with or without TGF-β1, 48 hours later QRT-PCR were performed to determine the mRNA expression of *DNMT1* and *DNMT3A*. (D) KYSE30 cell were treated with TGF-β1 at indicated concentrations or for defined intervals, and KYSE450/KYSE150 cells were treated with anti-TGF-β antibody (5ng/ml) for three days, then expressions of DNMT1 and DNMT3A were determined by Western blots. KYSE30 cells transfected with siRNA against *DNMT1* (E) or *DNMT3A* (F), and then treated with or without TGF-β1, 48 hours later QRT-PCR and Western Blots were performed to determine the expression of TIP30; *P < 0.05. Each bar represented the mean ±sd. of samples measured in triplicate, and each experiment was repeated at least three times.

To investigate the effects of TGF-β1 on *TIP30* methylation, we treated ESCC cells with TGF-β1 or anti-TGF-β antibody before extracting the genomic DNA and subjecting it to MSP analysis. The results showed that TGF-β1 induced a decrease in unmethylated promoter alleles and an increase in methylated promoter alleles in KYSE30 cells (Fig. 3A, left). In contrast, anti-TGF-β antibody suppressed *TIP30* methylation in KYSE450 and KYSE150 cells (Fig. 3A, right). The decreased expression of TIP30 by TGF-β1 was independent of canonical TGF-β1 signaling, since siRNA-mediated knockdown of Smad3 did not restore TIP30 expression upon TGF-β1 treatment (Fig. 3B). The results confirmed that TGF-β1 silences TIP30 expression by inducing promoter methylation.

To further explore the mechanisms by which TGF-β1 promotes methylation of *TIP30*, we analyzed expression of DNMTs. The mRNA expression of DNMT1 and DNMT3A were unchanged after TGF-β1 treatment (Fig. 3C). However, the protein expression of DNMT1 and DNMT3A were increased upon TGF-β1 treatment in both dose-dependent and time-dependent manners in KYSE30 (Fig. 3D, left and middle). In contrast, the anti-TGF-β antibody decreased DNMT1 and DNMT3A expression in KYSE450 and KYSE150 (Fig. 3D, right). To confirm the roles of DNMT1 or DNMT3A on TIP30 expression, specific siRNA against DNMT1 and DNMT3A were used. The results showed that the silencing of DNMT1 or DNMT3A restored TIP30 expression in TGF-β1 treated cells (Fig. 3E and F). These data suggest that TGF-β1 promotes methylation of *TIP30* by increasing expression of DNMT1 and DNMT3A.

### TIP30 inhibits TGF-β1-induced EMT and tumor metastasis

**Figure 4:**
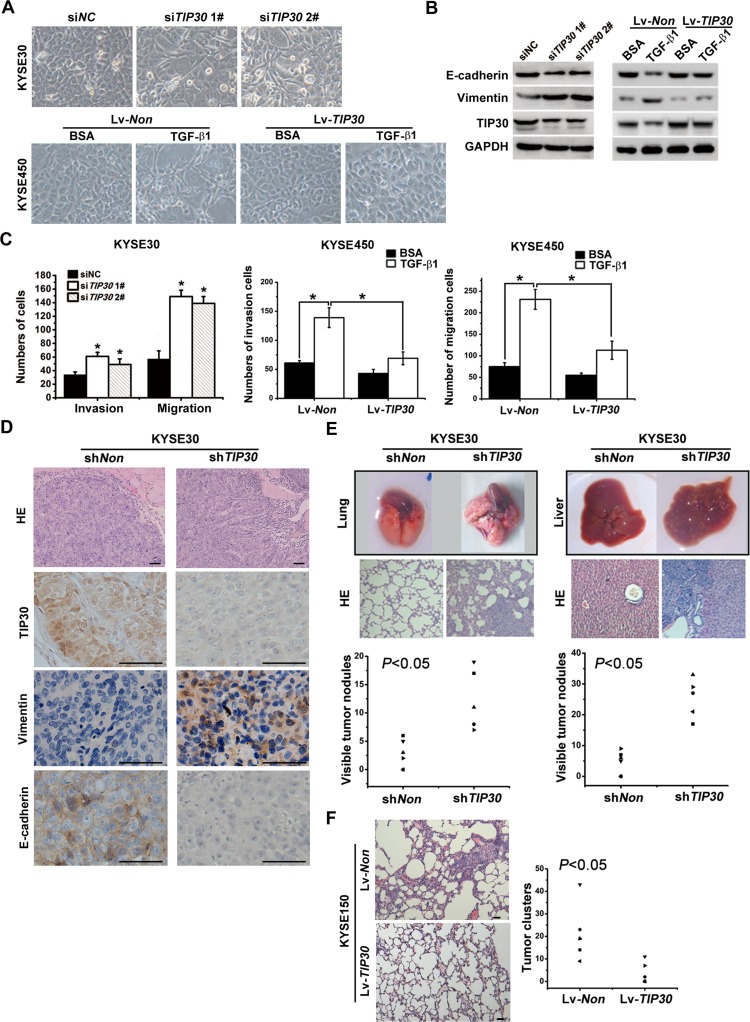
TIP30 inhibited TGF-β1-induced EMT and tumor metastasis KYSE30 cells transfected with siRNA against *TIP30* for 48 hours; KYSE450-*TIP30* cells and KYSE450-*Non* cells were treated with or without TGF-β1 for 48 hours; (A) morphologies were shown by phase-contrast microscopy (magnification, × 200); (B) the expressions of E-cadherin, Vimentin and TIP30 were determined by Western Blots; (C) invasion and migration assay were performed, total number of invaded and migrated cells were quantified and compared to the control samples; error bar indicate SD, *P < 0.05. (D) Male Balb/c nude mice were injected subcutaneously with KYSE30-sh*TIP30* cells or KYSE30-sh*Non* cells into the right flank of each animal. HE-staining and IHC staining for TIP30, Vimentin and E-cadherin on tumors sections, scale bar: 50μm. (E) Male Balb/c nude mice were injected intravenously with 1×10^6^ KYSE30-sh*TIP30* cells or KYSE30-sh*Non* cells through the tail vein, and then lung metastasis (left) and liver metastasis (right) were evaluated. The upper, metastatic nodules on the surface of the lung and liver are shown; the middle, representative H&E staining of lung and liver are shown; the lower, the numbers of nodules were quantified and values for each group are denoted (*P < 0.05, Student's t test). (F) Male Balb/c nude mice were injected subcutaneously with KYSE150-*TIP30* cells or KYSE150-*Non* cells into the right flank of each animal. Representative lung tissue sections by HE-staining from each group were shown in the left. Scale bar: 50μm. The number of lung metastatic foci in each group was calculated under microscope in the right (*P < 0.05, Student's t test).

The effects of TIP30 in TGF-β1-regulated EMT and metastatic potential were determined both *in vitro* and *in vivo*. KYSE30 cells were transfected with siRNA against *TIP30*. Silencing TIP30 led to morphologic changes, from square-like epithelial to spindle-like mesenchymal phenotype (Fig. 4A upper). Moreover, E-cadherin expression was decreased, whereas the vimentin expression was increased in the TIP30-depleted cells (Fig. 4B left and Supplemental Fig. 1A). EMT phenotype was also observed in KYSE30 cells stably expressing *TIP30* short hairpin RNA (shRNA) (Supplemental Fig. 1B and C). On the other hand, KYSE450 cells were infected with the Lv-*TIP30* or Lv-*Non* control virus and then treated with TGF-β1 or BSA for 48h. Overexpression of TIP30 blocked TGF-β1-induced EMT as determined by examination of cell morphology, expression of E-cadherin and Vimentin (Fig. 4A lower and Fig. 4B right). Migration and invasion assay showed that knockdown of *TIP30* resulted in a clear and potent migrative and invasive phenotype in KYSE30 cells (Fig. 4C left). In the contrast, overexpression of TIP30 may reduce TGF-β1-induced increases in cell invasion and migration (Fig. 4C middle and right). All of these results demonstrate that decrease of TIP30 plays an important role in TGF-β1-induce EMT in ESCC cells.

KYSE30 cells were infected with lentivirus expressing sh*Non* or sh*TIP30* to establish KYSE30-sh*Non* and KYSE30-sh*TIP30* cells respectively. Cells were then injected into the flanks of nude mice (n=6 per group). Compared to non-specific RNA interference, knockdown of *TIP30* significantly enhanced the growth and invasion of tumor cells (Supplemental Fig. 2) Furthermore, H&E staining and IHC staining for TIP30, Vimentin and E-cadherin were performed on tumor sections. A clear boundary between the tumor and its adjacent non-tumor tissue was often observed in KYSE30-sh*Non* cells generated tumors. However, irregular tumor invasion with a decreased E-cadherin expression and an increased Vimentin expression were observed in tumors induced by KYSE30-sh*TIP30* cells (Fig. 4D). The *in vivo* data confirms our *in vitro* observations that a loss of TIP30 promotes the cell invasion and EMT in ESCC.

To further elucidate the inhibitory effects of TIP30 on tumor metastasis, both experimental and spontaneous metastasis assays were used. KYSE30-sh*TIP30* and KYSE30-sh*Non* cells were intravenously injected into nude mice. After 8 weeks, the mice were euthanized and the lungs and livers were harvested. The number of metastatic nodules on the surface of the lungs and livers was significantly higher in mice injected with KYSE30-sh*TIP30* cells than in mice injected with KYSE30-sh*Non* cells (Fig. 4E, lower). Histological studies confirmed that the lesions were caused by extravasation and subsequent tumor growth of KYSE30 cells into the lungs and livers (Fig. 4E, middle). Meanwhile, KYSE150 cells infected with Lv-*TIP30* or Lv-*Non* control virus were injected subcutaneously into each mouse. Spontaneous metastasis assays showed that overexpression of TIP30 significantly reduced the incidence of lung metastases in nude mice (Fig. 4F). Since the tumors displayed severe liquefactive necrosis, the volume was not detected. All the above revealed that TIP30 negatively regulates tumor metastasis in ESCC *in vivo*.

### TIP30 blocked β-catenin signaling activated by TGF-β1

**Figure 5:**
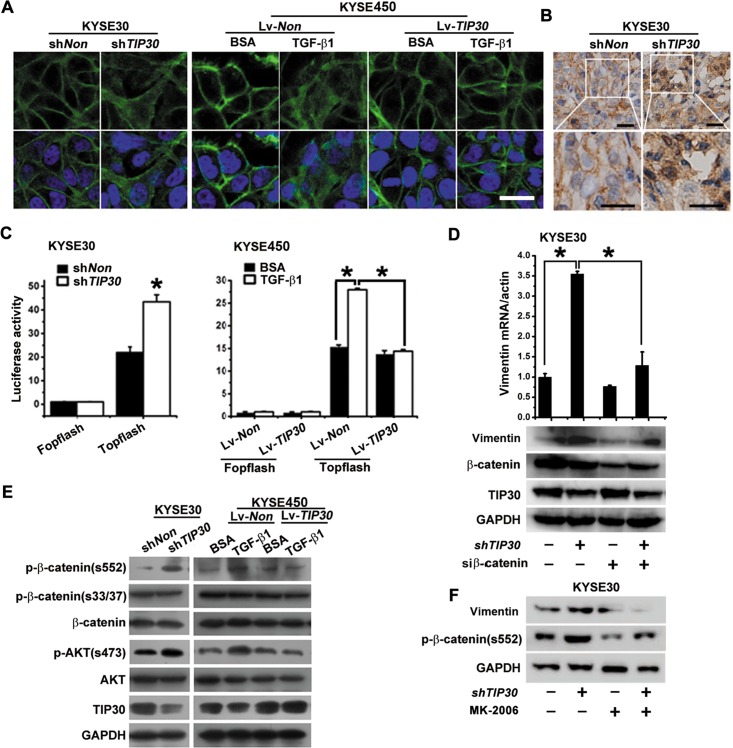
TIP30 blocked β-catenin signaling activated by TGF-β1 (A, C and E) KYSE30 cells were infected with Lv-sh*TIP30* to downregulated *TIP30* expression; KYSE450 cells were infected with Lv-*TIP30* to overexpression *TIP30*, three days later cells were treated with or without TGF-β1 for 48 hours. (A) IF analysis of β-catenin were performed. The green signals represent the staining of β-catenin and the blue signal represents the DAPI-stained nuclei; scale bar: 20μm. (B) After 4 weeks' orthotopic xenograft transplantation of KYSE30-sh*TIP30* cells or KYSE30-sh*Non* cells in nude mice, tumors derived from each group were immunostained for β-catenin, scale bar: 50μm. (C) TOP-Flash reporter gene assays were used to determine the transcriptional activity of β-catenin (error bar indicate SD, * *P*<0.05). (D) KYSE30-sh*TIP30* cells or KYSE30-sh*Non* cells were transfected with siRNA against β-catenin, 72 hours later expression of Vimentin were detected by QRT-PCR and Western Blots (error bar indicate SD, * *P*<0.05). (E) Phosphorylation levels and total expression levels of β-catenin and AKT were determined by Western Blots using specific antibodies. (F) KYSE30-sh*TIP30* cells or KYSE30-sh*Non* cells were treated with MK-2206, 48 hours later expression of Vimentin and p-β-catenin (s552) were detected by Western Blots (* *P*<0.05). Data shown represent three different experiments.

β-catenin has long been used as a marker for EMT. It not only functions as a major component of cell-cell adhesion, both also mediates transcriptional activation. A high-magnification picture of β-catenin staining showed downregulation of TIP30 promoted β-catenin to become disassociated from cell contacts and become translocated into the cytosol. Overexpression of TIP30 inhibits TGF-β1-induced redistribution of β-catenin (Fig. 5A). Consistent with the *in vitro* experimental results, cytosol and nucleus staining of β-catenin were found in KYSE30-sh*TIP30* cells generated tumors, while the control tumors showed a membranous staining of β-catenin (Fig. 5B). Because the cytoplasmic relocalization of β-catenin results in an increased pool of the protein that is able to move to the nucleus and stimulate transcription [22], we examined the transcriptional activation of the β-catenin reporter, TOP-flash. As expected, TIP30 silence sharply increased β-catenin-dependent transcriptional activity. Conversely, overexpression of TIP30 suppressed TGF-β1-induced activation of β-catenin (Fig. 5C). Previous study shows that β-catenin targets the Vimentin and ZEB1 genes for their transcription during EMT [23, 24]. In our study, TIP30 regulates Vimentin expression but not ZEB1 in ESCC (Supplemental Fig. 1A). To confirm the importance of β-catenin in increased expression of Vimentin induced by TIP30 silence, siRNA against β-catenin was used. The results showed TIP30 silence-induced Vimentin expression was significantly blocked in β-catenin-depleted cells (Fig. 5D). To underlie the regulatory mechanism of β-catenin activity by TIP30, the phosphorylation status of β-catenin was examined via western blots with indicated antibodies (Fig. 5E). GSK-3β mediated phosphorylation of S33/37 did not change upon *TIP30* depletion or TGF-β1 treatment. However, AKT mediated phosphorylation of S552 was significantly increased after *TIP30* depletion and TGF-β1 treatment. Furthermore, TGF-β1 mediated β-catenin phosphorylated at S552 was blocked by TIP30 overexpression. The results indicate that TIP30 inhibits phosphorylate of β-catenin at S552 and suppresses activation of β-catenin. Recent reports suggest that a TIP30 protein complex regulates EGFR endocytosis, resulting in the inhibition of AKT activation [25, 26]. Phosphorylation and activity of AKT were also detected, as shown in Fig. 5E. TIP30 silence significantly increased AKT activation, and overexpression of TIP30 inhibits TGF-β1 mediated AKT activation. Furthermore, blockage of AKT by MK-2206 attenuated TIP30 decrease-induced activation of β-catenin and upregulation of Vimentin (Fig. 5F). Together, we conclude that decreased expression of TIP30 facilitates TGF-β1-mediated AKT/β-catenin activation.

### Reduced TIP30 expression predicted a poor prognosis in ESCC patients

**Figure 6:**
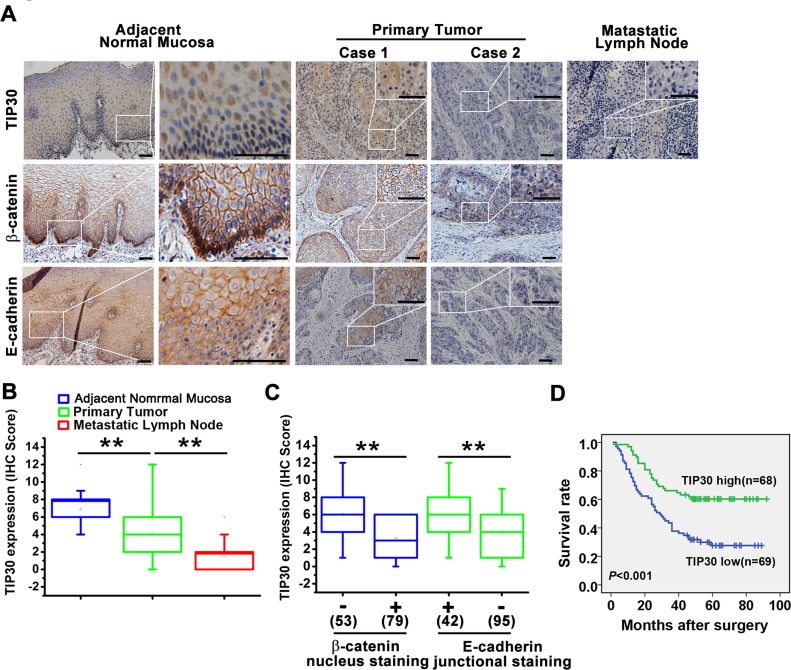
TIP30 expression correlated with EMT status in human ESCC, and is indicative of patient survival (A) Representative images of TIP30, β-catenin and E-cadherin in primary tumor tissue, the adjacent normal mucosa and metastatic lymph node examined by IHC. Scale bar: 50μm. (B) Comparisons of TIP30 expression level among different groups, including the adjacent normal mucosa, primary tumor tissue and metastatic lymph node (Mann-Whitney U test: **P < 0.01). (C) The relationships between TIP30 expression and protein expression of β-catenin and E-cadherin were analysed (Mann-Whitney U-test: **P<0.01). Nuclear β-catenin negative n=53 and nuclear β-catenin positive n=79; junctional E-cadherin positive n=42 and junctional E-cadherin negative n=95. Spread of data denoted by box-and-whisker plot, the central box represents the value from the lower to upper quartile (25th–75th percentile). The middle line represents the median. The whisker ends 1 and 99 percentiles. (D) Kaplan-Meier survival analysis of OS in ESCC patients based on TIP30 expression. Differences in survival between TIP30 low expression group and TIP30 high expression group were evaluated by the log-rank test.

The expression of TIP30 was examined by IHC in 137 primary tumors and paired adjacent normal mucosa, as well as 62 metastatic lymph nodes (Fig. 6A). In the normal epithelial cells, TIP30 showed intense staining mainly in the nucleus just above basal cell layer. In the tumor cells, TIP30 staining was mainly detected in the cytoplasm and significant weaker than that in the normal epithelial cells. Mann-Whitney U test revealed that the immunostaining score of TIP30 was highest in the adjacent normal mucosa and lowest in the metastatic lymph nodes (Fig. 6B).

On the basis of the TIP30 expression in tumor cells, 137 patients with ESCC were divided into two groups: the high-expression (n =68) and low-expression group (n = 69). The correlations of TIP30 expression and clinicopathologic features were further analyzed. TIP30 expression was inversely associated with the depth of tumor invasion (*P*=0.002), lymph node metastasis (*P*=0.042) and advance stage (*P*=0.002). However, there was no significant correlation between TIP30 expression with age, gender, tumor location, tumor differentiation or distant metastasis (Table 1).

**Table 1:** The associations of *TIP30* expression with clinicopathological characteristics in ESCC patients

	Total	Low expression	High expression	*P* value
Age (means.±d. years)	59.48±8.68	59.65±8.74	59.33±8.75	NS.
Gender				NS.
Male	83	44	39	
Female	54	25	29	
Tumor location				NS.
Upper	2	2	0	
Mid-thoracic	86	41	45	
Lower	49	26	23	
Histlogy				NS.
Well-Moderate	108	50	58	
Poor	29	19	10	
pT				0.002
pT_1-2_	81	32	49	
pT_3-4_	56	37	19	
pN				0.042
pN_0_	25	8	17	
pN_1_	112	61	51	
pM				NS.
pM_0_	113	56	57	
pM_1_	24	13	11	
TNM Stage				0.002.
I-II	81	32	49	
III-IV	56	37	19	

s.d. =standard deviation; NS, not significant; TNM, tumour, node, metastasis;

Moreover, we also detected β-catenin and E-cadherin expression levels in the ESCC tissues and paired adjacent normal mucosa (Fig. 6A), in the normal epithelial cells, β-catenin and E-cadherin showed unequivocal and strong membranous staining just above basal cell layer, whereas, in the basal cell layer, β-catenin located mainly in the nucleus. In the tumor tissues, 59.8% (79/132) had nucleus positive staining of β-catenin and 69.3% (95/137) had aberrant E-cadherin protein expression with negative staining or less than 70% membranous staining. We found loss of TIP30 correlated with nuclear β-catenin, and aberrant E-cadherin expression (*P*<0.001, Mann-Whitney U test, Fig. 6C).

The potential associations between TIP30 expression and overall survival (OS) evaluated in the 137 patients included in the present study. Log-rank test showed that ESCC patients with low TIP30 expression experienced poor overall survival (OS) than patients with high TIP30 expression (median survival time, 29 months vs. 49 months; *P* < 0.001; Fig. 6E). By univariate analysis, tumor differentiation, TNM stage and TIP30 low expression were prognostic factors for OS. Nevertheless, multivariate analysis showed that downregulation of TIP30 and tumor differentiation were 2 independent prognostic predictors for ESCC patients enrolled in this study (Supplemental Table 1). Thus, decreased expression of TIP30 may serve as a prognostic indicator for patients with ESCC.

## DISCUSSION

TGF-β1, a potent EMT inducer present in the tumor microenvironment, is involved in the metastasis and progression of many types of carcinomas including esophageal carcinoma. TIP30 is a putative tumor metastasis suppressor and its expression and function in ESCC remains unknown. This study reveals a novel unique modulatory role of TIP30 in TGF-β-mediated EMT and tumor metastasis in human esophageal cancer.

Here we found that TIP30 expression was negatively correlated with TGF-β1 in ESCC cells. TGF-β1 decrease TIP30 expression levels in both dose-dependent and time-dependent manners. So far, we know less about the regulation mechanism of TIP30. Previous studies have suggested that the expression of TIP30 could be activated by JAK/STAT3 pathway or suppressed by DNA methylation and mir-10b. It was reported that Sorafenib, a tyrosine kinase inhibitor (TKI), decreased expression of TIP30 via JAK/STAT3 signaling in HCC [27]. In PDAC, TIP30 is a direct target of mir-10b. A typical CpG island is found in the promoter of *TIP30* and *TIP30* is frequently metylated in hepatocarcinoma and breast cancer [15, 16]. We examined methylation status of *TIP30* in ESCC cell lines and ESCC patients to find that *TIP30* is also frequently hypermethylated in both ESCC cell lines and ESCC patients. Furthermore, the methylation statutes of *TIP30* are positively correlated with the levels of TGF-β1 in ESCC cell lines. A recent study suggested that TGF-β1 also plays an important and controversial role in the regulation of DNA methylation. TGF-β1 may increase DNMT1/3A expression and cause nuclear translocation of DNMT1/3A in prostate cancer and kidney fibrogenesis [28, 29]. TGF-β1 may also decrease expression of DNMT1/3B in HCC cells [30]. DNMT1 has been implicated primarily in the maintenance of methylation patterns that occurs during cellular replication and preferentially methylates hemimethylated DNA[31]. We demonstrate that *TIP30* can be methylated by TGF-β1 via up-regulation of DNMT1 and DNMT3A. TGF-β1 did not change the mRNA expression of DNMT1 and DNMT3A, but increased their protein level directly, this may be the reason that methylation and downregulation of TIP30 occurred at early time phases. However, it remains unclear how TGF-β1 regulates DNMTs protein levels. It has reported posttranslational modifications of acetylation and ubiquitination, as well as regulation of micoRNA were involved in expression regulation of DNMTs. The regulation of TIP30 expression by TGF-β1 implies a potential role of TIP30 in TGF-β1-mediated biological functions.

During cancer progression, TGF-β1 frequently switches from tumor suppressor to tumor promoter [32, 33]. Oncogenic signals may blunt TGF-β-induced growth arrest and apoptosis, while enhancing TGF-β-induced pro-invasive and pro-metastatic responses [11, 34, 35]. This may involve a change in the balance between canonical SMAD signaling and non-SMAD signaling [12]. Our results shows that in ESCC cells, TGF-β1 induces a typical EMT phenotype accompany with increased cell invasion and migration. Further, we found down-regulation of TIP30 may also initiate EMT and increase properties of metastasis, but over-expression of TIP30 may partially inhibit TGF-β1-induced EMT and pro-metastatic responses. The results indicate that TGF-β1-induced EMT and pro-metastatic responses were mediated partially by silence of TIP30. We also demonstrate that TIP30 may inhibit phosphorylation and activation of β-catenin. Our data shows that silence of TIP30 facilitates TGF-β1-mediated activation of AKT/β-catenin signaling, which subsequently induces EMT and pro-metastatic responses.

Our findings present the inhibition effects and the underlying mechanism of TIP30 in tumor metastasis and EMT, and also provide new insights into the molecular mechanisms of TGF-β1-mediated tumor progression.

## MATERIALS AND METHODS

### Cell culture and lentiviral infection

ESCC cell lines (YES2, KYSE30, KYSE450, KYSE150, KYSE180 and KYSE410) were cultured at 37°C in an atmosphere containing 5% CO2 in RPMI 1640 medium supplemented with 10% fetal bovine serum. A normal esophageal cell line Het-1A was cultured in LHC-9 medium supplemented with 2% fetal bovine serum. Beijing Microread Gene Technology Co, Ltd. used short tandem repeat profiling to authenticate KYSE30, KYSE150 and KYSE450 cell lines on Nov. 2013. All the cells were also periodically authenticated by morphologic inspection and tested for Mycoplasma contamination. TGF-β1 and TGF-β antibody treatment were performed at the indicated concentration (TGF-β, Peprotech; TGF-β antibody, clone 1D11, R&D Systems). 5-Aza-2_-Deoxycytidine (5-Aza-2_dC) (Sigma-Aldrich) treatment was performed using 10uM of 5-Aza-2_dC for 3 days. TIP30 depletion and overexpression experiments were performed by infection of cells with shRNA-expression or *TIP30*-expression lentivirus using 293T cells as packaging cell lines, as described previously [17]. Sequence of siRNA designed against TIP30, DNMT1, DNMT3A, Smad3 and β-catenin can be obtained from Supplemental Table 2.

### RNA extraction and reverse transcription PCR

Total RNA was isolated using Trizol reagent (Invitrogen). First-strand cDNA was generated using the PrimeScript RT reagent Kit (TAKARA). Analysis of mRNA levels was performed on a 7500 Fast Real-Time PCR System (Applied Biosystems) with SYBR Green-based real-time PCR. Actin was used as an endogenous control to normalize the amount of total RNA in each sample. The primer sequences can be obtained from Supplemental Table 3.

### Western Blots

Total cell lysate was prepared in 1×SDS buffer. Proteins at the same amount were separated by SDS-PAGE and transferred onto PDVF membranes. After probing with individual antibodies, antigen–antibody complex was visualized by enhanced chemiluminescence's reagents Supersignal (Pierce Biotechnology). The primary antibodies specific against E-cadherin, Vimentin, DNMT1, DNMT3A, p-S552-β-catenin and p-S33/37-β-catenin were from Cell Signaling Technology (Beverly, MA); the β-catenin antibody was from BD Biosciences (SanJose, CA), the GAPDH antibody was from Kangchen Biothechnology Company (Guangzhou, P.R. China); the TIP30 antibody was described previously [17].

### Immunofluorescence staining

Cells were grown on glass chamber slides fixed with 4% paraformaldehyde/PBS. permeabilized with 0.2% Triton X-100/PBS, and blocked with 10% goat serum in PBS. Cells were incubated with primary antibodies against β-catenin (1:500) (BD Biosciences) at 4°C overnight, then incubated with a goat anti-mouse Alex Flour 488 antibody(Invitrogen) and stained with DAPI(Invitrogen). All matched samples were photographed (control and test) a confocal laser-scanning microscope (FLUOVIEW FV-1000).

### DNA Extraction and Methylation-Specific PCR (MSP) Analysis

Genomic DNA was extracted from 5×10^6^ cells or 10 mg tissue using QIAamp DNA Mini Kit (Qiagen). Genomic DNA was treated with sodium bisulfite has been described [36]. DNA (5 μg) was denatured in 33 μl of 0.3 mol/L NaOH at 37°C for 15 minutes, without using restriction endonuclease. Denatured DNA was mixed directly with 333 μl of bisulfite solution and treated in darkness. The bisulfite solution was prepared as either 2.4 mol/L sodium metabisulfite (pH 5.0–5.2) (Sigma-Aldrich)/0.5 mmol/L hydroquinone (Sigma-Aldrich) for a 4-hour treatment 31 or 3.1 mol/L sodium bisulfite (pH 5.0–5.2) (Sigma-Aldrich)/0.5 mmol/L hydroquinone for a 16-hour treatment. DNA was desalted and purified using the QIAEX II Gel Extraction Kit (Qiagen). DNA was then treated with 0.3 mol/L NaOH at 37°C for 15 minutes and precipitated with 3 mol/L ammonium acetate and 3 volumes of ethanol. Recovered DNA was purified again then dissolved in 20–50 μl of TE buffer (pH 8.0) and stored at −20°C. MSP was performed using *Taq* Gold polymerase (Applied Biosystems) as described previously. MSP products were subcloned into pGEM-T Vector (Promega) and transformed into Escherichia coli. Candidate plasmid clones were sequenced by Huada Scientific (Beijing, P.R. China).

### Quantification of TGF-β1

ESCC cells were cultured in fresh serum-free media for 24 hours, and then total TGF-β1 in the cell culture supernatant was measured by specific enzyme-linked immunosorbent assay (ELISA) (Peprotech). TGF-β1 concentration was normalized to number of cells, determined by cell counting with a hemocytometer. Data are expressed as pg/ml of TGF-β1 per 10^5^ cells.

### Transwell Migration and Invasion Assay

Transwell migration assays were quantified *in vitro* using Transwell chambers with polycarbonate membrane filters (8 μm pore size; Corning) according to the manufacturer's instructions. In brief, the lower chamber was filled with 0.6 ml medium containing 20% fetal bovine serum and 0.2ml of medium that contained 3×105 cells under serum-starving conditions was plated in the upper chamber and incubated at 37°C for 48 hours. Then cells that had not migrated were removed from the upper face of filters using cotton swabs. The cells that migrated through the membrane and attached to the bottom of the membrane were fixed and stained with crystal violet. Images of five random fields were captured from each membrane and the number of migratory cells was counted, and the extent of migration was expressed as the average number of cells per microscopic field at a magnification of 100. The mean of triplicate assays for each experimental condition was used. Similar inserts coated with Matrigel (Chemicon) were used to determine invasive potential in invasion assay. Two independent investigators were blinded when reading the migration and invasion assays.

### TOP-flash luciferase assays

ESCC cells were cotransfected with pTOP-flash or pFOP-flash and Renilla vector (pRL-TK) using Lipofectamine 2000 (Invitrogen) in triplicate. After 24 hours cells were treated with or without 5 μM of TGF-β1 for 48 hours. The cells were then lysated and analyzed with the Dual Luciferase Kit (Promega) on a Centro XS 3 LB 960 (Berthold Technology GmbH & Co KG). pTOP-flash and pFOP-flash plasmids were gifts from Dr. Mingzhou Guo (Department of Gastroenterology & Hepatology, Chinese PLA General Hospital, Beijing, China).

### Animal experiments

Male Balb/c nude mice at 6 week-old were housed under standard conditions and cared for according to the institutional guidelines for animal care. All animal experiments were approved by the Institutional Animal Care and Use Committee (IACUC) of PLA General Hospital. For the tumorigenicity assay, KYSE30 cells were infected with LV-sh*TIP30* and LV-sh*Non* at MOI 20 and 5×10^6^ cells were injected subcutaneously into each mouse (n = 6 mice / group). The mice were monitored over the course of the experiment and euthanized for histopathology examination at day 28 after cells inoculation. The tumors volume was calculated according to the formula: V =length × width2 × 0.5. For experimental metastasis assay, each experimental group (sh*TIP30* and sh*Non*) consisted of 5 mice. Briefly, 1 × 106 cells were injected intravenously through the tail vein into each mouse. After 8 weeks, the mice were euthanized, the presence of tumor nodules was macroscopically determined and the number of tumor nodules formed on the lung/liver surfaces was counted. The lungs and livers were excised and embedded in paraffin for histopathology examination. For the spontaneous metastasis assay, KYSE150 cells were infected with LV-*TIP30* and LV-*Non* at MOI 20 and 5×10^6^ cells were injected subcutaneously into each mouse (n = 6 mice / group). After 8 weeks, mice were euthanized and examined for development of pulmonary metastasis under microscope.

### Patient samples and Immunohistochemical staining

A total of 93 tissue specimens, including 8 cases of normal mucosa and 85 cases of ESCC, used in MSP analysis were acquired from the Pathology Department of the Medical College of Shantou University (Shantou, P.R.China), collected from 2007 to 2009. This study was approved by the ethical committee of the Tumor Hospital of Shantou University Medical College. The tissue specimens used in IHC staining had been removed from 137 patients with ESCC who had undergone surgery at The 150 hospital of Chinese PLA (Luoyang, Henan, P.R.China) between 2001 and 2005. Patient consent and approval from the 150 Hospital of Chinese PLA Ethics Committee was obtained in order in advance for research purposes. Cancer sample was taken from the tumor tissue where there was no hemorrhage or putrescence, whereas the matched normal mucosa was taken from the surgical cutting edge, which was approximately 3–5 cm away from the cancerous lesion. The expression of TIP30, β-catenin and E-cadherin proteins in the specimens was detected with an immunohistochemistry assay. Details of Immunohistochemistry were described previously [17].

Evaluation of immunostaining was independently conducted by 2 experienced pathologists. The expression of TIP30 was scored according to the signal intensity and distribution, details described previously [18]. Tissues with immunohistochemical scoring ≤4 were considered as low expression and with scoring 5-12 as high expression. The expression of TIP30 was evaluated according to the percentage of stained nuclei, independently of intensity. Positive staining was defined as 20% or greater nucleic staining of the population of cells examined [37]. E-cadherin expression was scored as either normal (junctional) or aberrant, as previously described. Aberrant staining was defined as either negative staining or less than 70% membranous staining of the population of cells examined. Normal staining was defined as 70% or greater membranous staining of the study population [38].

### Statistic Analysis

The analyses were carried out using SPSS 16.0 for Windows software (Chicago, IL). *P*-values for dichotomous variables were two-tailed and based on the Pearson chi-square test or the Pearson chi-square test with continuity correction. Continuous variables were analyzed with a Student's t test. Survival curves were calculated using Kaplan–Meier method, and analysis was carried out with the log-rank test. Patient follow-ups were completed on March 15, 2011. The median follow-up period was 41 months (range, 1–93 months). Univariate and multivariate analyses were based on the Cox proportional hazards regression model. Data were presented as the mean ± SEM. A value of *P* <0.05 was considered statistically significant.

## SUPPLEMENTARY MATERIAL FIGURES AND TABLES



## Abbreviations

ESCC: esophageal squamous cell carcinoma
QRT-PCR: quantitative reverse-transcription polymerase chain reaction
shRNA: short-hairpin RNA
EMT: epithelial–mesenchymal transition
MSP: Methylation-Specific PCR
BGS: bisulfite genomic sequencing
IF: Immunofluorescence
DAPI: 4′,6-diamidino-2-phenylindol
IHC: Immunohistochemical
OS: overall survival
