# Supplementary material for: TGF-β1 induces epigenetic silence of TIP30 to promote tumor metastasis in esophageal carcinoma
Source: Oncotarget. 2014 Dec 3;6(4):2120–33. doi: 10.18632/oncotarget.2940 (PMC4385840; doi:10.18632/oncotarget.2940)
Supplement: Supplementary file 1 [file oncotarget-06-2120-s001.pdf]

TGF-β1 induces epigenetic silence of TIP30 to promote tumor metastasis in esophageal carcinoma

Supplementary Material

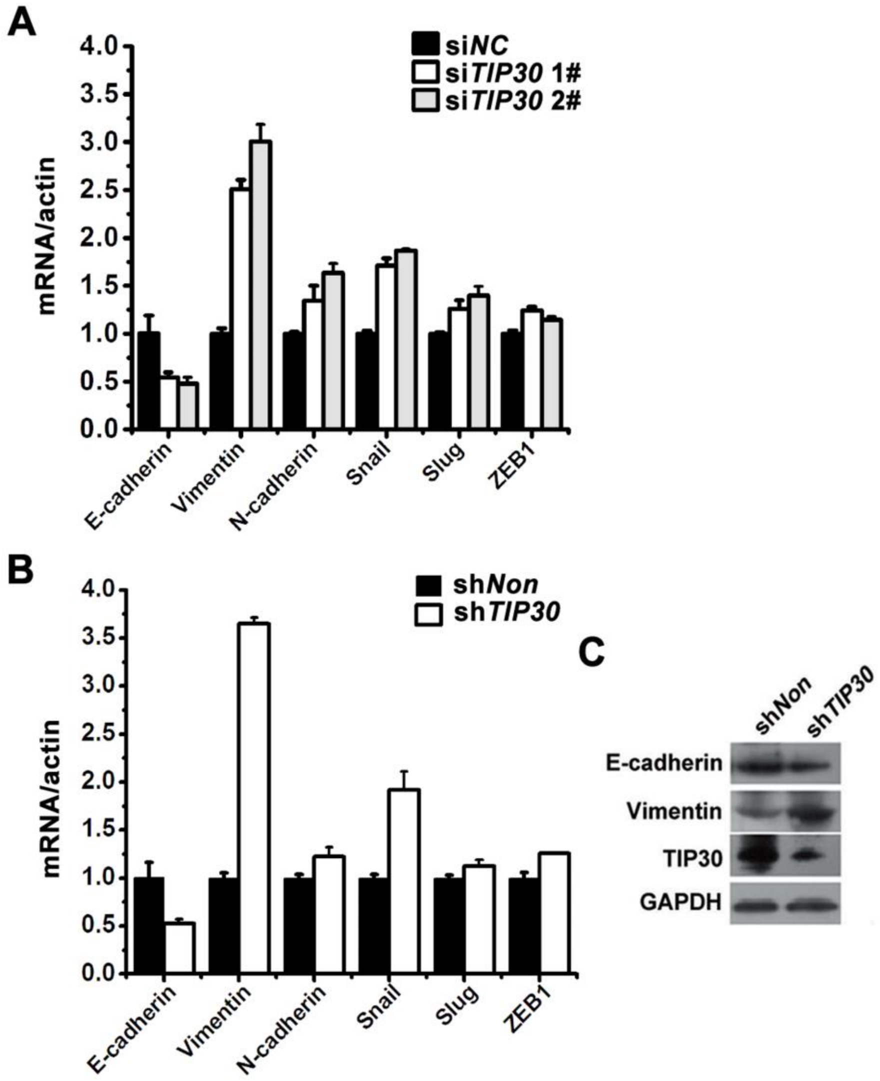

Supplemental Figure 1: Knockdown of TIP30 promoted EMT. (A) KYSE30 cells transfected with siRNA against *TIP30* for 48 huors; (B) KYSE30 cells infected with Lv-sh*TIP30* or Lv-shNon for 72 hours, expression of E-cadherin, Vimentin, N-cadherin, Snail, Slug and ZEB1 were detected by QRT-PCR. (C) Expression of E-cadherin, Vimentin and TIP30 in KYSE30-sh*TIP30* cells and KYSE30-sh*Non* cells were confirmed by Weatern Blots.

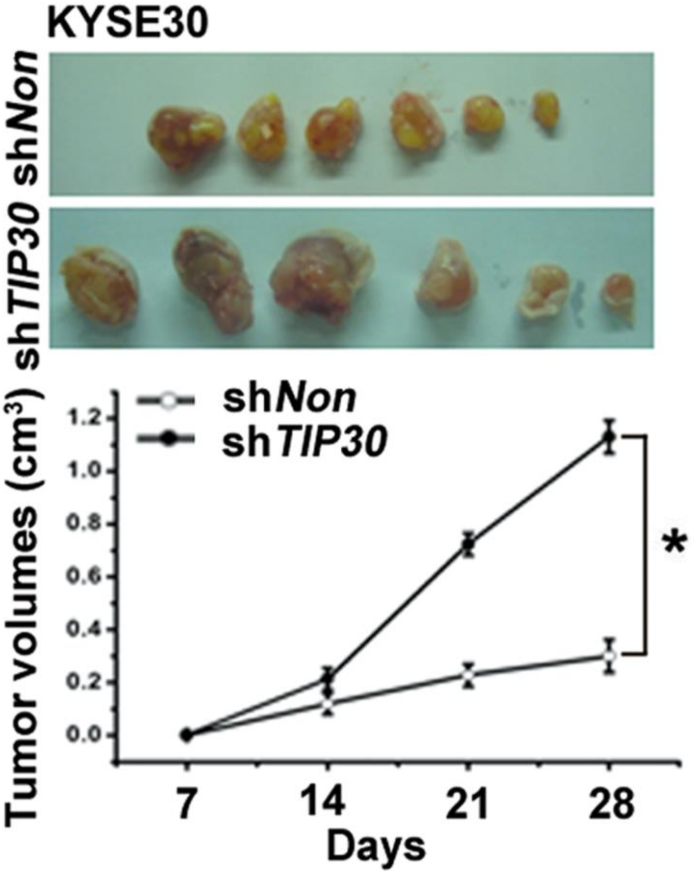

Supplemental Figure 2: Knockdown of TIP30 promoted invasive growth of tumor. Male Balb/c nude mice were injected subcutaneously with KYSE30-sh*TIP30* cells or KYSE30-sh*Non* cells into the right flank of each animal. Imaging of each tumor 28 days after subcutaneous injection were shown (upper); tumor size was monitored and recorded every 7 days (lower, data are the means ± SD of the tumor volumes derived from each group, *$P < 0.05$).

Supplemental Table 1 Univariate and multivariate analyses of factors associate with disease-specific survival in patients with ESCC.

Variables	Hazard ration	95% CI	*P*
Univariate analysis			
Age (< median vs.≥median)	1.19	0.947-1.496	0.136
Gener (female vs. male)	1.262	0.991-1.608	0.060
Differentiation (well and Moderate vs. poor)	1.388	1.078-1.788	0.011
pT factor (T_{1-2} vs. T_{3-4})	1.251	0.999-1.567	0.051
pN factor (pN_0 vs.pN_1)	1.108	0.814-1.508	0.515
pM factor(pM_0 vs.pM_1)	1.051	0.792-1.394	0.731
TNM stage (Ⅰ-Ⅱ vs.Ⅲ-Ⅳ)	1.263	1.009-1.583	0.042
TIP30 expression (low vs. high)	0.419	0.261-0.670	<0.001
E-cadherin (Aberrant vs. normal)	0.642	0.382-1.079	0.095
Multivariate analysis			
TIP30 expression (low vs. high)	0.444	0.276-0.714	0.001
Differentiation (well and moderate vs. poor)	1.229	1.006-1.676	0.045

Multivariate analysis, Cox proportional hazards regression model. Variables were adopted for their prognostic significance by univariate analysis and no obvious correlation between each other.

Supplemental Table 2 siRNA Sequence used in these studies

siRNA	Sequence (sense strand)
TIP30 1#	GCAGAAUAAAUCCGUCU
TIP30 2#	GGAGGGAUUUGUUCGUG
DNMT1 1#	GGAGCUGUUCUUGGUGGAU
DNMT1 2#	CCGUAGCCCUGGAAACAAA
DNMT3A 1#	CAGUGGUGUGUGUUGAGAAGCUGAU
DNMT3A 2#	CGGCUCCAGAUGUUCUUCGCUAAUA
Smad3 1#	CAGCACAUAAUAACUUGGACCUGCA
Smad3 2#	CACCAGGAUGCAACCUGAAGAUCUU
β-catein	CAGGGGGUUGUGGUUAAGCUCUU
NC	UUCUCCGAACGUGUCACGUUU

NC: negative control

Supplemental Table S3: Sequence of primers for PCR

Primers for QRT-PCR	
TIP30	F: GAAGACTCGGTGAGCATGCG
	R: GCGATACGCTCTGAGCCAGT
E-cad	F: TGCCCAGAAAATGAAAAAGG
	R: GTGTATGTGGCAATGCGTTC
N-cad	F: ACAGTGGCCACCTACAAAGG
	R: CCGAGATGGGGTTGATAATG
Vim	F: GAGAACTTTGCCGTTGAAGC
	R: GCTTCCTGTAGGTGGCAATC
Fn1	F: CAGTGGGAGACCTCGAGAAG
	R: TCCCTCGGAACATCAGAAAC
Snail1	F: AATCGGAAGCCTAACTACAGCG
	R: GTCCCAGATGAGCATTGGCA
Slug	F: GGGGAGAAGCCTTTTTCTTG
	R: TCCTCATGTTTGTGCAGGAG
ZEB1	F: CAGCTTGATACCTGTGAATGG
	R: TATCTGTGGTCGTGTGGGACT
DNMT1	F: CCTGAGGCCTTCACGTTCAA
	R: ACTTGTGGGTGTTCTCAGGC
DNMT3A	F: TATTGATGAGCGCACAAGAGAGC
	R: GGGTGTTCCAGGGTAACATTGAG
Primers for MSP	
TIP30-M	F: TTTTCGGGGTTATTTTTTTCGC
	R: CACCTTCCCTAATCGAACCG
TIP30-U	F: GTTTTTGGGGTTATTTTTTTTGT
	R: CACCTTCCCTAATCAAACCA

QRT-PCR, Quantitative reverse-transcription polymerase chain reaction; MSP: Methylation-Specific PCR; F, forward; R, reverse.
